# Quantitative Trait Locus Analysis of Protein and Oil Content in Response to Planting Density in Soybean (*Glycine max* [L.] Merri.) Seeds Based on SNP Linkage Mapping

**DOI:** 10.3389/fgene.2020.00563

**Published:** 2020-06-25

**Authors:** Xiaocui Tian, Kaixin Zhang, Shulin Liu, Xu Sun, Xiyu Li, Jie Song, Zhongying Qi, Yue Wang, Yanlong Fang, Jiajing Wang, Sitong Jiang, Chang Yang, Zhixi Tian, Wen-Xia Li, Hailong Ning

**Affiliations:** ^1^Key Laboratory of Soybean Biology, Ministry of Education, Northeast Agricultural University, Harbin, China; ^2^Key Laboratory of Soybean Biology and Breeding/Genetics, Ministry of Agriculture, Northeast Agricultural University, Harbin, China; ^3^Soybean Research Institute, Northeast Agricultural University, Harbin, China; ^4^State Key Laboratory of Plant Cell and Chromosome Engineering, Institute of Genetics and Developmental Biology, Chinese Academy of Sciences, Beijing, China

**Keywords:** soybean, protein and oil content, density, gene, density response

## Abstract

Soybean varieties suitable for high planting density allow greater yields. However, the seed protein and oil contents, which determine the value of this crop, can be influenced by planting density. Thus, it is important to understand the genetic basis of the responses of different soybean genotypes to planting density. In this study, we quantified the protein and oil contents in a four-way recombinant inbred line (FW-RIL) soybean population under two planting densities and the response to density. We performed quantitative trait locus (QTL) mapping using a single nucleotide polymorphism (SNP) linkage map generated by inclusive composite interval mapping. We identified 14 QTLs for protein content and 17 for oil content at a planting density of 2.15 × 10^5^ plant/ha (D1) and 14 QTLs for protein content and 20 for oil content at a planting density 3.0 × 10^5^ plant/ha (D2). Among the QTLs detected, two oil-content QTLs was detected at both plant densities. In addition, we identified 38 QTLs for the responses of protein and oil contents to planting density. Of the QTLs detected, 70 were identified in previous studies, while 33 were newly identified. Fourty-five QTLs accounted for over 10% of the phenotypic variation of the corresponding trait, based on 23 QTLs at a marker interval distance of ~600 kb detected under different densities and with the responses to density difference. Pathway analysis revealed four candidate genes involved in protein and oil biosynthesis/metabolism. These results improve our understanding of the genetic underpinnings of protein and oil biosynthesis in soybean, laying the foundation for enhancing protein and oil contents and increasing yields in soybean.

## Introduction

Soybean (*Glycine max* [L.] Merri.) is a major source of vegetable oil and feed protein. Dry soybean seeds are composed of approximately 40% protein and 20% oil (Rajcan et al., 2005). Increasing the protein content (PC) and oil content (OC) of soybean seeds is an important breeding objective.

The protein and oil contents of seeds are inherited as quantitative traits controlled by multiple genes, leading to a low efficiency of soybean improvement based on phenotypic selection. Therefore, much research has focused on quantitative trait locus (QTL) mapping for protein and oil content. To date, 248 QTLs for protein content and 327 for oil content have been deposited in SoyBase (http://www.soybase.org). Most of this research was carried out using conventional molecular marker techniques (Powell et al., 1996), such as restriction fragment length polymorphism (RFLP), amplified fragment length polymorphism (AFLP), and simple sequence repeat (SSR) mapping, which has reduced the accuracy of QTL mapping and led to reduced genetic stability due to the low densities of these markers and their uneven distributions in the genome. In recent years, single nucleotide polymorphism (SNP) markers have become powerful tools for exploring plant genomes due to their high density, good genetic stability, and suitability for accurate, high-throughput genotyping (Lee et al., 2015; Zhang et al., 2018). SNPs markers have been successfully used in soybean research. For example, Liu et al. (2017) used parental Zhong huang × Hua xia3 hybridization RIL populations obtained using SNP markers and detected four QTLs for protein content and nine QTLs for oil content in two environments in 2 years. Akond et al. (2014) detected one QTL for protein content and 11 QTLs for oil content using an F_5:8_ RIL population derived from MD 96-5722 × Spencer using 5376 SNP markers. Wang et al. (2015) used an RIL population from V97-1346 × R05-4256 and detected 13 SNPs for protein content. In these studies, QTL mapping for protein and oil content in soybean plants was conducted using bi-parental populations. This technique has several limitations, as it results in the detection a small number of QTLs and limits the richness of the alleles and phenotypic variation. However, more recently, four-way recombinant inbred line (FW-RIL) populations have been used for QTL mapping, which overcomes the limitation posed by populations with a narrow genetic basis. In addition, these lines contain four alleles in a single locus, which greatly improves the QTL detection capacity. Moreover, high-density linkage maps can be constructed using FW-RIL populations, and the genetic markers are highly polymorphic (Ning et al., 2016; Liu et al., 2019).

In recent decades, the planting density of soybean has gradually been increasing, which has greatly affected the quality of soybean seeds. Bellaloui et al. (2014) investigated the effects of planting density on the composition of soybean seeds and observed that the highest protein and the highest oil contents occurred at different planting densities. Akond et al. (2012) detected two QTLs for protein and 6 QTLs for oil content at a higher planting density (25 cm row spacing) and three QTLs for oil content at a lower plant density (50 cm row spacing). By comparing the phenotypes and QTLs detected, many different genetic bases for protein and oil content at different planting densities have been uncovered. It is therefore important to breed plant varieties that are suitable for various planting densities and to analyze the genetic basis of the effects of planting densities on protein and oil content.

In the current study, we analyzed the genetic basis of the responses of protein and oil contents in soybean to different planting densities based on phenotypic data from a 144 member FW-RIL population and high-density SNP maps. We identified 45 QTLs with phenotypic variance explained (PVE) values of >10% under different planting densities and with the responses to density difference. Finally, we identified four candidate genes that might control protein and oil content under the influence of planting density that could be useful to improve molecular breeding and increase the protein and oil contents of soybean.

## Materials and Methods

### Plant Materials

Four soybean varieties, Kenfeng 14 (protein content 39.69% and oil content 20.34%), Kenfeng 15 (protein content 38.68% and oil content 22.76%), Heinong 48 (protein content 44.74% and oil content 19.05%), and Kenfeng 19 (protein content 42.52% and oil content 19.26%), were used as parents to construct an FW-RIL population. Kenfeng 14, Kenfeng 15, Heinong 48, and Kenfeng 19 were obtained by crossing Suinong10 × Changnong 5, Suinong 14 × Kenjiao 9307, Hefeng 25 × (Kenfeng 4 × Gong 8861-0), and Ha 90-6719 × Sui 90-5888, respectively (Table S1). Two F_1_ seeds were harvested by crossing Kenfeng 14 × Kenfeng 15 and Heinong48 × Kenfeng 19 in 2008. The FW-F1 populations were obtained by crossing plants from two F1 seeds in 2009. From 2010 to 2012, FW-F1 seeds were planted in Harbin, China in the summer and in Hainan in the winter. The plants were self-crossed for six generations to obtain a stable FW-RIL population using the single-seed descent method. The 144 resulting FW-RILs were used in the experiments.

### Field Experiment and Trait Measurements

The entire experiment was conducted in 2015 and 2016. In 2015, the test sites were Harbin (45°43′N, 126°45′E) (E1) and Keshan (48°18′ N, 126°15′ E) (E2); in 2016, the test sites were Acheng (45°32′N, 126°58′E) (E3), Shuangcheng (45°22′ N, 126°18′ E) (E4), and Harbin (E5). The field experiments were arranged in a split-plot design with three replications. The main treatment comprised a normal planting density of 2.15 × 10^5^ plants/ha (D1) and an increased planting density of 3.0 × 10^5^ plants/ha (D2), which were implemented using a plant spacing of 0.07 and 0.05 m, respectively, in each row of a three-ridge block 3 meters long and 2.1 meters wide. The four parents and FW-RILs were planted in a subplot with a random permutation. The environmental conditions, fertility, year, planting date, planting location, annual rainfall, and annual accumulated temperature are summarized in Table S2. Field management was performed using local field cultivation conditions for soybean.

Five mature plants obtained from the four parental lines and the FW-RIL population were randomly selected from the middle row of each plot. Protein and oil content were measured using an INFRAC TM1214 near-infrared grain quality analyzer (Foss). The detailed process is described in Zhang et al. (2018). The average values of three samples were used as the phenotypic values of the parents and FW-RILs.

### Statistical Analysis

#### Estimating the Effect of Density

The response to density (RD) value refers to the change in the quality of a trait due to an increase in the planting density. RD was calculated as the remainder of the trait value at high density (D2) after subtracting the trait value at normal density (D1). Specifically, RD was estimated using the conditional variable method (Zhu, 1995) with the following formula:

$$\begin{array}{l} RD=y_{D2}-C_{D1D2}\left(y_{D1}-{\bar{y}}_{D1}\right)/V_{D1} \end{array}$$

where *RD* is the response to density difference, *y*_*D*1_ is the phenotypic value of the first (normal) density, *y*_*D*2_ is the phenotype value of the second (high) density, C_*D*1*D*2_ is the covariance between phenotypes of quality traits under the two densities, and ${\bar{y}}_{\text{D}1}$ and *V*_*D*__1_ are the average and variance, respectively, of quality traits under the first (normal) density.

The maximum, minimum, standard deviation, range, skewness, and kurtosis of the protein and oil content for each density in each environment were also calculated. Analysis of variance (ANOVA) was conducted to compare the phenotypic values of protein and oil contents at each planting density in each environment or jointly in multiple environments and in response to density difference.

Similar to the method used to estimate average heritability in multiple macro-environments, the average broad-sense heritability (*h*^2^) at multiple planting densities was estimated using the following equation:

$$\begin{array}{l} h^{2}=\frac{\sigma_{G}^{2}}{\sigma_{G}^{2}+\frac{\sigma_{GD}^{2}}{d}+\frac{\sigma^{2}}{erd}} \end{array}$$

where *h*^2^ is broad-sense heritability, $\sigma_{G}^{2}$ is the variance of the genotype, $\sigma_{GD}^{2}$ is the genotype × density interaction, σ^2^ is the variance of error, *e* is the number of environments, *d* is the number of planting densities, and *r* is the number of replications. The data for protein and oil content were analyzed using Proc MIXED in SAS 9.2 statistical software (SAS Institute, Cary, NC, US).

#### SNP Genotyping

DNA was extracted from the fresh leaves of plants from the FW-RIL population from the cross (Kenfeng 14 × Kenfeng 15) × (Heinong 48 × Kenfeng 19) using the CTAB method (Doyle and Doyle, 1990). The DNA was used for SNP genotyping analysis with the SoySNP660K BeadChip at Beijing Boao Biotechnology Co., Ltd. The SNP markers were filtered for minor allele frequency (MAF > 0.05), with a maximum of <10% missing sites per SNP (Belamkar et al., 2016). A linkage map of soybean containing 2332 SNP markers (https://figshare.com/s/4a7b8caea2c29f891bc3) was constructed using GAPL 1.2 software (http://www.isbreeding.net/software/), the length range covered is 3539.66 cM on the soybean genome.

#### QTL Analysis

Based on the SNP linkage map, the average protein and oil contents under each planting density and RD in every environment were used to map the QTLs with the inclusive composite interval mapping (ICIM) method (Zhang S. et al., 2017) using GAPL V1.2. Firstly, The LOD (likelihood of odds) score for putative QTLs was determined after 1,000 permutations at a significant level of *P* < 0.05 with objective to find major QTL. Then mapping QTL was re-analyzed by setting LOD score of 3 to screen minor QTL. The QTLs were named as follows: q—trait name—chromosome name—sequence number, where q represents QTL, PC, and OC represent protein content and oil content, respectively, and RD represents the response to density. The QTLs that were mapped to the same marker region were given the same sequence number.

#### Identification of Candidate Genes for Protein and Oil Content

Twelve QTLs and eleven QTLs with PVE > 10% were detected within a 600 kb genomic region under different planting densities and with the responses to density difference, respectively. To further explore whether these QTLs are related to protein and oil content in soybean, we attempted to identify the candidate genes associated with the QTLs. We used the *Glyma.Wm82.a2.v1* gene model in SoyBase (https://soybase.org/SequenceIntro.php) to identify all gene sequences based on the intervals of each QTLs with PVE > 10%. As a result, various highly expressed genes that controlled protein and oil content were identified based on the BAR website (http://www.bar.utoronto.ca). Finally, we used the KEGG website (http://www.kegg.jp/blastkoala/) to identify candidate protein and oil content-related genes based on KEGG pathway analysis results.

## Results

### Phenotypic Variation

**Figure 1:**
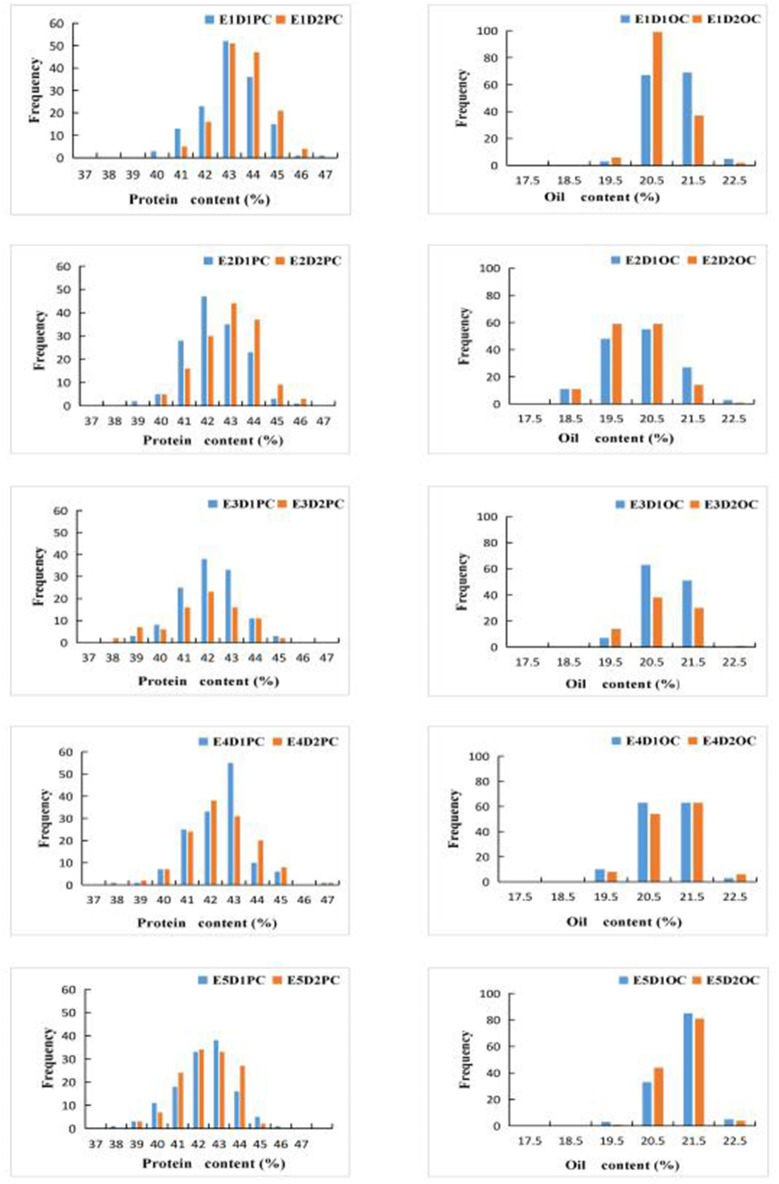
Bar charts of protein content (PC) and oil content (OC) values in the FW-RIL population. E1: Harbin (2015), E2: Keshan (2015), E3: Acheng (2016), E4: Shuangcheng (2016), E5: Harbin (2016), D1: the first (lower) planting density (2.15 × 10^5^ plants/ha), D2: the second (higher) planting density (3 × 10^5^ plants/ha).

We constructed bar chats of the protein and oil contents of 144 FW-RILs (Figure 1). Protein and oil contents at different planting densities in the same environment exhibited a continuous normal distribution. Analysis of the protein and oil contents of the four parents at different densities located in the interval between the minimum and maximum values of the FW-RIL population (Table 1) indicated that the protein and oil contents of the FW-RILs was significantly higher than those of the parents, suggesting that transgressive inheritance was involved in protein and oil contents in the FW-RILs. Most of the kurtosis and skewness values of the data were between −1 and 1, suggesting that the population was suitable for ANOVA of related traits. An F test revealed significant (*P* < 0.01) differences in quality traits among the densities in a single environment. Therefore, the FW-RILs were constructed from two high-oil (Kenfeng14, Kenfeng15) and two high-protein varieties (Kenfeng19, Heinong48), providing an ideal basis for QTL analysis.

**Table 1:** Variation analysis of protein content (PC) and oil content (OC) for parental lines and the FW-RIL population under two planting densities in each environment.

	**Parents**									**FW-RILs**												
**Environment^a^**	**D1^b^**				**D2^b^**				**D1^b^**						**D2^b^**						***F*^f^**	***h^**2**^***
	**KF14^c^**	**KF15^c^**	**HN48^c^**	**KF19^c^**	**KF14^c^**	**KF15^c^**	**HN48^c^**	**KF19^c^**	**Min**	**Max**	**Mean**	**Std^d^**	**Skew**	**Kurt**	**Min**	**Max**	**Mean**	**Std^e^**	**Skew**	**Kurt**		
**PC^e^**																						
2015HRB	42.9	41.1	39.9	43.9	43.8	42.9	45.3	44.7	39.8	46.8	42.64	1.19	0.15	0.49	40.8	46	43.08	1.04	−0.01	0.09	1.65^**^	0.31
2015KS	43	40.9	39.5	43.3	42.7	41.2	42.2	43.5	38.15	45.99	41.88	1.22	0.02	0.84	39.26	45.84	42.4	1.25	0.05	−0.15	1.59^**^	0.43
2016AC	39.65	40.75	42.5	42.45	39.2	40	42.6	42.6	38.2	45	41.65	1.24	−0.14	−0.11	37.6	45	41.43	1.64	−0.32	−0.25	1.68^**^	0.5
2016SC	41.3	42.35	43.05	41.45	41.8	42.05	41.7	42.4	38	46.1	41.93	1.3	−0.07	0.62	38.5	46.2	41.97	1.37	0.12	0.07	1.54^**^	0.54
2016HRB	40.8	41.35	41.6	42.45	41.9	41.4	42	42.95	38	45.4	41.82	1.39	−0.36	0.01	38.4	44.8	41.89	1.31	−0.31	−0.48	1.76^**^	0.5
**OC^e^**																						
2015HRB	20.37	20.27	21.88	19.82	19.77	20.25	19.6	20.26	18.74	22.3	20.5	0.55	0.27	1.51	18.96	21.82	20.27	0.48	0.28	0.69	1.10^**^	0.1
2015KS	19.08	19.85	20.97	19.19	19.41	18.71	19.45	19.82	17.58	22.22	19.71	0.87	0.06	−0.41	17.74	21.56	19.52	0.75	0.17	−0.43	1.81^**^	0.12
2016AC	20.55	20.46	19.65	19.79	20.09	21.54	19.54	19.83	18.53	21.39	20.36	0.57	−0.68	0.49	18.66	21.67	20.25	0.65	−0.28	−0.37	1.87^**^	0.45
2016SC	20.4	20.54	20.5	20.68	20.65	20.28	19.66	20.51	18.85	21.8	20.37	0.58	−0.2	−0.15	18.79	22.11	20.49	0.64	−0.19	0.23	2.33^**^	0.32
2016HRB	20.47	20.33	21.07	20.81	20.55	20.27	20.58	20.74	18.57	22.31	20.69	0.58	−0.69	1.92	18.69	21.83	20.67	0.46	−0.37	2.16	1.92^**^	0.23

a *2015HRB, Harbin in 2015; 2015KS, Keshan in 2015; 2016AC, Acheng in 2016; 2016SC, Shuangcheng in 2016; 2016HRB, Harbin in 2016*.

b *D1, the first (lower) planting density (2.15 × 10^5^ plants/ha), D2, the second (higher) planting density (3 × 10^5^ plants/ha)*.

c *KF14, Kenfeng14; KF15, Kenfeng15; HN48, Heinong48; KF19, Kenfeng19*.

d *Std, standard deviation*.

e *PC, protein content, OC, oil content*.

f** *significant at P < 0.01*.

**Figure 2:**
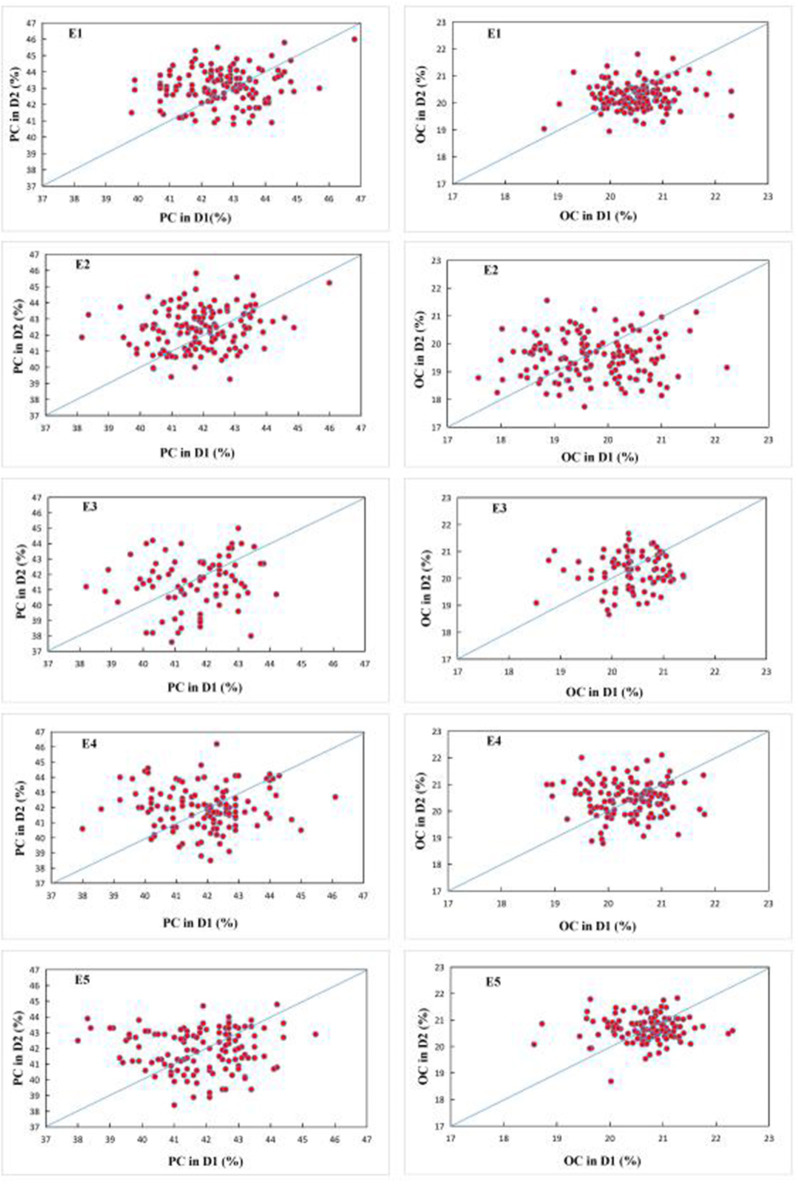
Q-Q plot of seed protein (left) and oil (right) content under two planting densities across five environments. Each circle represents one FW-RIL. E1: Harbin (2015), E2: Keshan (2015), E3: Acheng (2016), E4: Shuangcheng (2016), E5: Harbin (2016).

ANOVA of the values of the parents and FW-RILs indicated that the genotype and genotype × density interaction effect was significant (*P* ≤ 0.01; Table 2). Compared to direct density effects, the genotype × density interaction effect was significant, indicating that density affects protein and oil content indirectly by altering the genetic basis of quality formation and that different genotypes have different responses to density increase. The estimated broad-based heritability varied for protein and oil content at different planting densities in each environment, indicating that density influences the genetic basis of protein and oil content in soybean seeds (Table 2). The effect of environment on the response of protein and oil content to density was significant (*P* ≤ 0.01; Table 3), indicating that the response of protein and oil content to planting density differed depending on the environment. In addition, the effect of increased planting density on protein and oil content varied markedly in terms of both direction and magnitude according to genotype (Figure 2). Therefore, we analyzed the effects of QTLs on protein and oil contents at different planting densities.

**Table 2:** Joint ANOVA for protein content (PC) and oil content (OC) for the FW-RIL population across five environments.

**Source**	**DF**	**PC**					**OC**				
		**SS**	**MS**	**F**	**Pr>F**	**Variance component**	**SS**	**MS**	**F**	**Pr > F**	**Variance component**
Environment (E)	4	680.30	170.08	45.01	<0.0001	0	520.66	130.17	123.99	<0.0001	0
Planting density (D)	1	19.08	19.08	5.05	0.0247	0.0048	2.53	2.53	2.41	0.1208	0.002
Genotype (G)	143	2,095.62	14.65	3.88	<0.0001	0.34	363.47	2.54	2.42	<0.0001	0.045
G × D	143	900.74	6.30	1.67	<0.0001	0.17	222.15	1.55	1.48	0.0003	0.033
E × D	4	70.51	17.63	4.67	0.0009	0.038	18.14	4.54	4.32	0.0017	0.007
Error	2602	9,831.03	3.78			3.66	2,731.57	1.05			1.02
h^2^						0.62					0.47

**Table 3:** Variation analysis of protein content (PC) and oil content (OC) in the FW-RIL population in response to planting density.

**Source**	**DF**	**PC**					**OC**			
		**SS**	**MS**	**F**	**Pr > F**		**SS**	**MS**	**F**	**Pr > F**
Replication	2	66.50	6.65	1.55	0.12		17.61	1.76	1.66	0.09
Environment (E)	4	263.47	65.87	15.36	<0.0001		166.28	41.57	39.14	<0.0001
Genotype (G)	143	779.69	5.45	1.27	0.02		190.33	1.33	1.25	0.03
E × G	562	2380.55	4.24	0.99	0.56		660.99	1.18	1.11	0.09
Error	966	4141.34	4.29				1025.97	1.06		

### QTLs for PC and OC at Two Planting Densities

**Figure 3:**
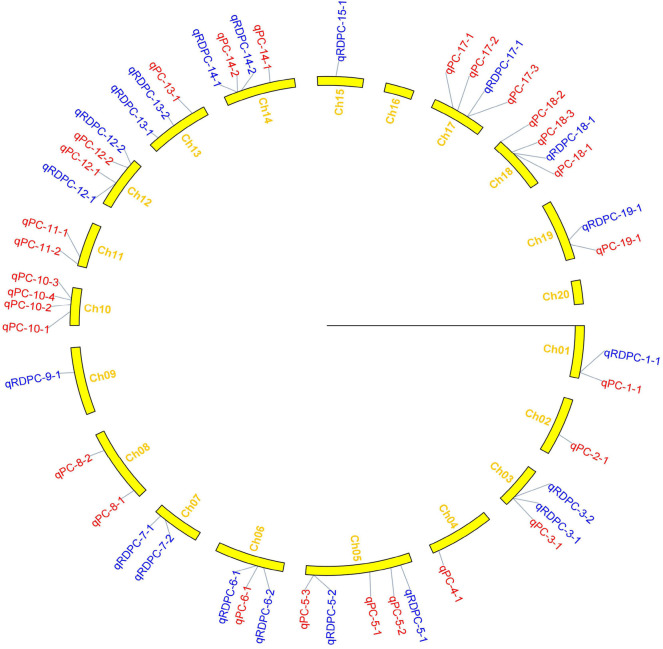
Distribution of QTLs for protein content on linkage groups detected in the soybean FW-RIL population. Red and blue represent QTLs detected for protein content under different planting densities and the response of density difference, respectively.

**Figure 4:**
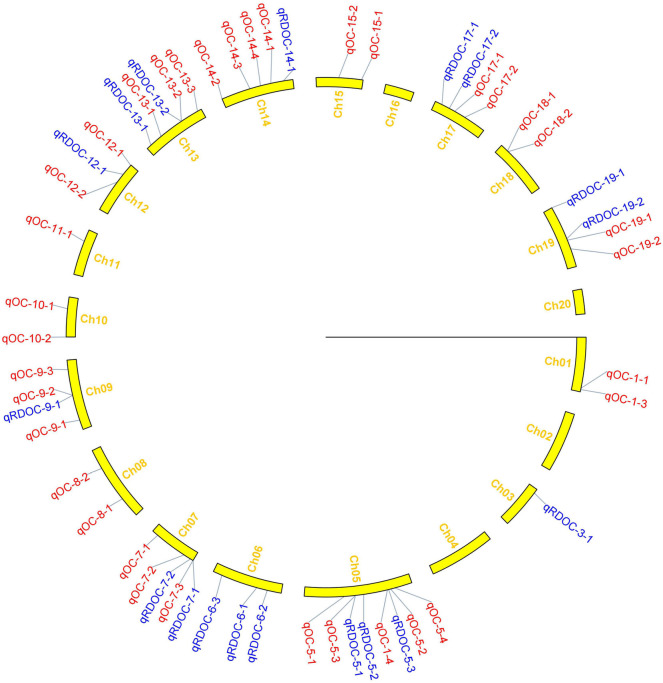
Distribution of QTLs for oil content on linkage groups detected in the soybean FW-RIL population. Red and blue represent QTLs detected for oil content under different planting densities and the responses to density difference, respectively.

**Figure 5:**
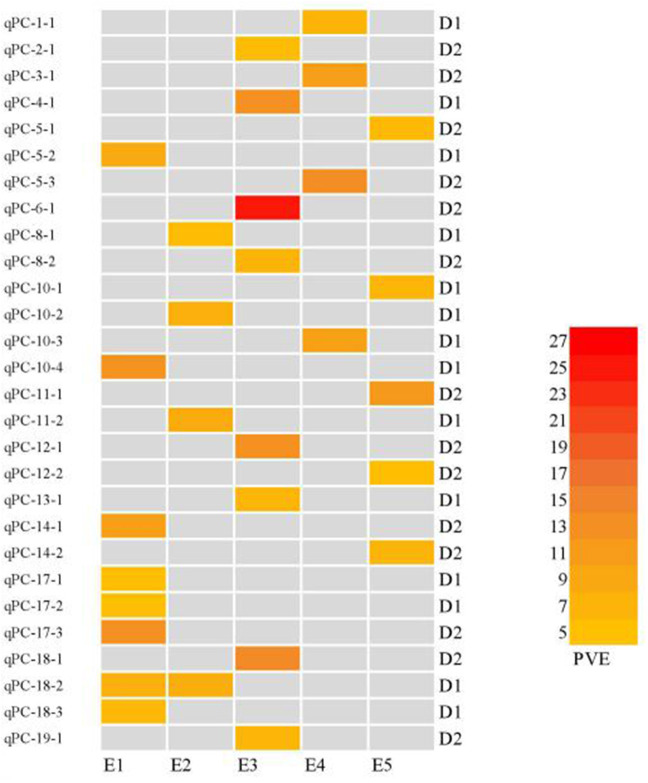
Heatmap of protein content QTLs at different planting densities with various PVE values higher than 10%. E1: Harbin (2015), E2: Keshan (2015), E3: Acheng (2016), E4: Shuangcheng (2016), E5: Harbin (2016). D1: the first (lower) planting density (2.15 × 10^5^ plants ha^−1^), D2: the second (higher) planting density (3 × 10^5^ plants ha^−1^).

**Figure 6:**
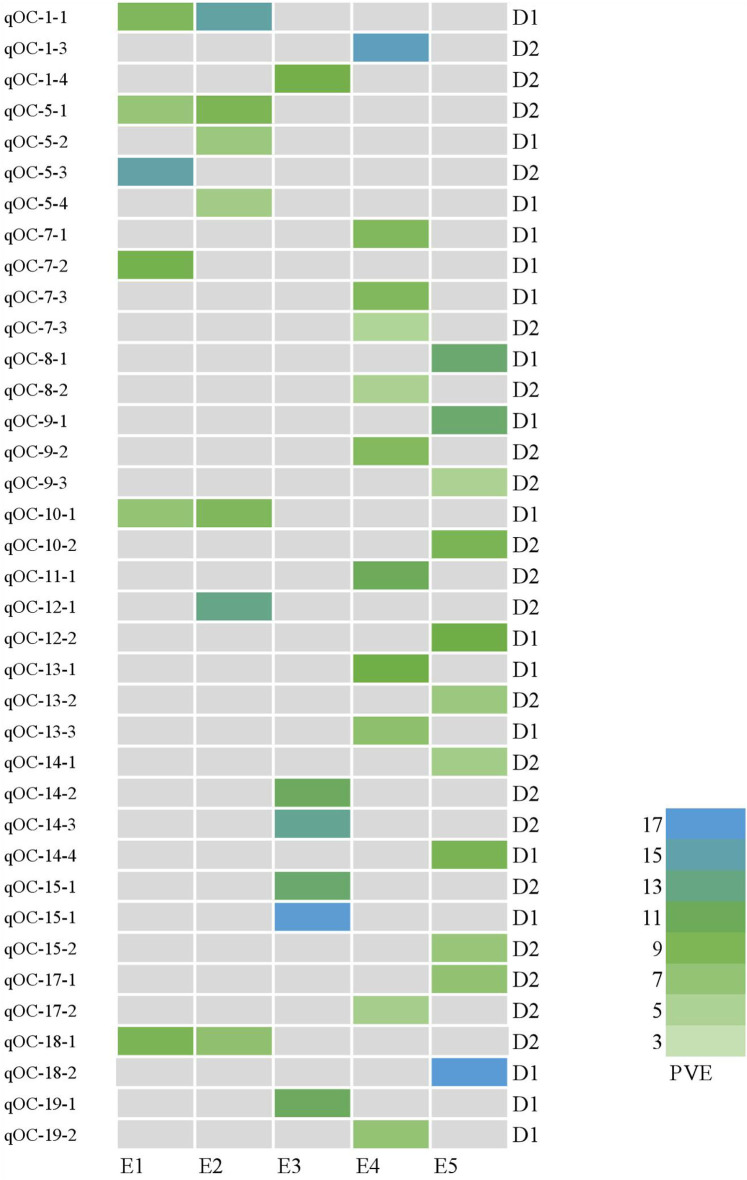
Heatmap of QTLs for oil content at different planting densities with various PVE values higher than 10%. E1: Harbin (2015), E2: Keshan (2015), E3: Acheng (2016), E4: Shuangcheng (2016), E5: Harbin (2016). D1: the first (lower) planting density (2.15 × 10^5^ plants ha^−1^), D2: the second (higher) planting density (3 × 10^5^ plants ha^−1^).

**Figure 7:**
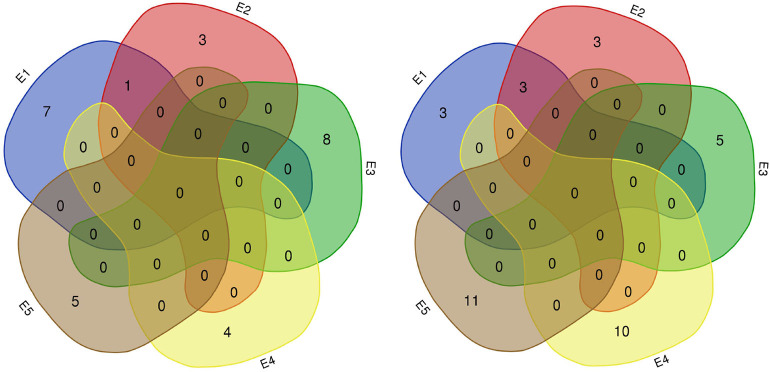
Number of QTLs for protein (left) and oil (right) content detected in five environments. E1: Harbin (2015), E2: Keshan (2015), E3: Acheng (2016), E4: Shuangcheng (2016), E5: Harbin (2016).

In this study, 65 QTLs associated with PC and OC were detected on 15 and 14 of the 20 soybean chromosomes under LOD value of 3 and under permutation method, respectively (Figures 3, 4), which 5 PC QTLs and 6 OC QTLs were located in two method. Of these, 28 PC QTLs (Table 4) and 37 OC QTLs (Table 5) were detected at different planting densities (D1 and D2). Among the QTLs detected, 14 for PC and 17 for OC were detected at D1. The LOD values ranged from 3.01 to 6.43. A single QTL accounted for 5.22% (qPC-17-2) to 14.58% (qOC-1-1) of phenotypic variance. The 14 remaining QTLs for PC and 20 QTLs for OC were detected at D2. The LOD values ranged from 3 to 8.93. The PVE values of the QTLs ranged from 4.77% (qOC-7-3) to 25.05% (qPC-6-1). Finally, 26 QTLs accounted for over 10% of the phenotypic variation. These findings indicate that protein and oil contents are controlled by both major-effect (PVE ≥ 10%) and minor-effect (PVE < 10%) QTLs (Figures 5, 6). Among these QTLs, only two QTLs (qOC-7-3, qOC-15-1) was simultaneously identified at both planting densities (Figure 7), indicating that the genetic basis for protein and oil content differed at different densities.

**Table 4:** QTLs for protein (qPC) identified under different planting densities in different environments.

**Trait**	**Planting density^a^**	**QTL**	**Method**	**Chromosome**	**Physical position (bp)**	**LOD**	**PVE (%)^b^**	**Add1^c^**	**Add2^c^**	**Add3^c^**	**Add4^c^**	**Environment^d^**	**References**
PC	D1	qPC-1-1	ICIM-3	1	41,354,793–43,754,056	3.01	7.06	0.23	−0.34	0.56	−0.46	E4	
		qPC-4-1	ICIM-3	4	16,292,606–41,180,931	4.22	12.67	−0.31	0.3	0.77	−0.76	E3	Stombaugh et al., 2004
		qPC-5-2	ICIM-3	5	3,379,706–3,450,946	4.63	8.82	−0.37	0.49	0.19	−0.31	E1	
		qPC-8-1	ICIM-3	8	276,792–15,554,288	3.46	5.64	−0.1	−0.25	0.6	−0.25	E2	Tajuddin et al., 2003; Reinprecht et al., 2006; Lu et al., 2013; Pathan et al., 2013
		qPC-10-1	ICIM-3	10	32,225,908–44,361,012	3.16	6.7	0.67	−0.46	0.29	−0.5	E5	Specht et al., 2001; Chen et al., 2007; Mao et al., 2013; Qi et al., 2014
		qPC-10-2	ICIM-3	10	44,570,190–45,647,371	3.12	7.67	0.57	−0.5	0.29	−0.36	E2	Chen et al., 2007
		qPC-10-3	ICIM-3	10	44,665,252–45,076,309	3.73	10.12	0.6	0.31	−0.3	−0.61	E4	Chen et al., 2007
		qPC-10-4	ICIM-3/ICIM-P	10	45,076,309–45,546,379	6.43	12.4/7.19	0.58/0.58	−0.54/−0.54	0.33/0.33	−0.37/−0.37	E1	
		qPC-11-2	ICIM-3	11	3,507,011–7,091,516	3.11	8.36	0.39	−0.76	0.06	0.31	E2	Brummer et al., 1997; Chapman et al., 2003; Lu et al., 2013; Mao et al., 2013
		qPC-13-1	ICIM-3	13	29,551,591–31,596,553	3.6	6.96	−0.66	0.01	0.26	0.39	E3	Mao et al., 2013
		qPC-17-1	ICIM-3	17	188,378–36,960,374	3.19	5.42	0.52	−0.5	0.02	−0.05	E1	Reinprecht et al., 2006; Mao et al., 2013; Wang et al., 2014b
		qPC-17-2	ICIM-3	17	34,150,351–35,150,689	3.31	5.22	0.55	−0.46	−0.06	−0.03	E1	Mao et al., 2013
		qPC-18-2	ICIM-3	18	6,442,474–6,652,232	4.32	7.72	−0.34	0.31	−0.38	0.41	E1	Mao et al., 2013
			ICIM-3			4.76	8.1	−0.6	0.46	−0.27	0.41	E2	
		qPC-18-3	ICIM-3	18	8,261,351–12,546,166	3.64	6.26	−0.53	0.13	0.1	0.31	E1	Reinprecht et al., 2006
PC	D2	qPC-2-1	ICIM-3	2	43,360,376–47,381,537	3.05	5.62	0.09	0.64	−0.43	−0.29	E3	Qi et al., 2014
		qPC-3-1	ICIM-3	3	1,393,672–1,560,122	3.13	10.47	0.3	0.29	0.13	−0.72	E4	
		qPC-5-1	ICIM-3	5	34,050,351–34,241,321	3.38	6.02	−0.14	−0.43	−0.01	0.58	E5	
		qPC-5-3	ICIM-3	5	30,826,149–40,096,192	3.82	13.25	0.44	−0.82	0.41	−0.03	E4	Jun et al., 2008; Pathan et al., 2013
		qPC-6-1	ICIM-3/ICIM-P	6	47,860,433–49,004,869	8.93	25.05/23.14	1.46/1.46	0.53/0.53	−0.99/−0.99	−1/−1	E3	Csanádi et al., 2001
		qPC-8-2	ICIM-3	8	3,050,275–3,253,888	3.28	6.99	0.36	−0.55	−0.29	0.49	E3	
		qPC-11-1	ICIM-3/ICIM-P	11	3,507,011–33,288,769	5.86	11.48/9.11	0.69/0.69	−0.11/−0.11	0.23/0.23	−0.82/−0.82	E5	Brummer et al., 1997; Chapman et al., 2003; Reinprecht et al., 2006; Gai et al., 2007; Lu et al., 2013; Mao et al., 2013; Wang et al., 2014b; Asekova et al., 2016
		qPC-12-1	ICIM-3/ICIM-P	12	16,850,384–16,953,996	5.11	12.6/11.64	0.46/0.46	−0.78/−0.78	−0.37/−0.37	0.69/0.69	E3	
		qPC-12-2	ICIM-3	12	2,151,310–2,257,434	3.12	5.42	−0.28	0	−0.31	0.59	E5	
		qPC-14-1	ICIM-3	14	13,471,993–16,178,681	3.00	10.3	0.56	−0.01	−0.37	−0.18	E1	
		qPC-14-2	ICIM-3	14	2,256,954–4,780,532	4.13	7.08	0.73	0	−0.19	−0.54	E5	Akond et al., 2014
		qPC-17-3	ICIM-3	17	39,258,351–39,557,300	3.96	12.75	−0.01	−0.38	−0.24	0.63	E1	Tajuddin et al., 2003; Warrington et al., 2015
		qPC-18-1	ICIM-3/ICIM-P	18	12,546,166–55,369,435	5.93	13.93/12.87	0.77/0.77	−0.74/−0.74	0.5/0.5	−0.54/−0.54	E3	Diers et al., 1992; Brummer et al., 1997; Jun et al., 2008; Eskandari et al., 2013; Lu et al., 2013; Mao et al., 2013
		qPC-19-1	ICIM-3	19	251,932–48,432,343	3.21	6.67	0.37	0.38	0.04	−0.79	E3	Diers et al., 1992; Mansur et al., 1996; Orf et al., 1999; Chapman et al., 2003; Tajuddin et al., 2003; Eskandari et al., 2013; Lu et al., 2013; Mao et al., 2013; Asekova et al., 2016

a *D1, the first (lower) planting density (2.15 × 10^5^ plants ha^−1^), D2: the second (height) planting density (3.0 × 10^5^ plants ha^−1^)*.

b *PVE, phenotypic variation explained*.

c *Add1, Add2, Add3, Add4, additive effects from Kenfeng14, Kenfeng15, Heinong48, Kenfeng19, respectively. Additive effect over than 0.05% are positive alleles*.

d *E1: Harbin in 2015; E2: Keshan in 2015; E3: Acheng in 2016; E4: Shuangcheng in 2016; E5: Harbin in 2016*.

**Table 5:** QTLs for oil content (qOC) identified under different planting densities in different environments.

**Trait**	**Planting density^a^**	**QTL**	**Method**	**Chromosome**	**Physical position (bp)**	**LOD**	**PVE (%)^b^**	**Add1^c^**	**Add2^c^**	**Add3^c^**	**Add4^c^**	**Environment^d^**	**References**
OC	D1	qOC-1-1	ICIM-3/ICIM-P	1	27,421,201–27,657,396	5.35	8.70/8.95	−0.14/−0.14	0.13/0.13	−0.25/−0.25	0.26/0.26	E1	Hyten et al., 2004
						5.35	14.58/8.86	−0.14/−0.14	0.13/0.13	−0.25/−0.25	0.26/0.26	E1	
		qOC-5-2	ICIM-3	5	3,379,706–3,450,946	3.11	6.45	0.09	−0.4	0.34	−0.04	E2	Wang et al., 2014b
		qOC-5-4	ICIM-3	5	2,209,578–4,202,110	3.02	5.9	−0.33	−0.28	0.65	−0.05	E2	Wang et al., 2014b; Han et al., 2015
		qOC-7-1	ICIM-3	7	1,974,268–2,303,275	3.96	8.59	0.07	−0.14	−0.19	0.25	E4	
		qOC-7-2	ICIM-3/ICIM-P	7	38,265,631–38,555,656	5.72	9.79/9.51	−0.14/−0.14	0.23/0.23	0.12/0.12	−0.21/−0.21	E1	Tajuddin et al., 2003
		qOC-7-3	ICIM-3	7	39,411,702–40,613,918	3.56	8.63	0.21	0.15	−0.08	−0.27	E4	Tajuddin et al., 2003
		qOC-8-1	ICIM-3	8	25,108,271–33,158,058	3.73	12.03	−0.23	−0.15	0.13	0.25	E5	Chen et al., 2007
		qOC-9-1	ICIM-3	9	3,352,797–4,194,469	3.77	11.83	−0.18	0.2	−0.2	0.19	E5	Mansur et al., 1996; Mao et al., 2013
		qOC-10-1	ICIM-3	10	44,665,252–45,076,309	4.2	7.11	−0.18	0.18	−0.16	0.16	E1	
						3.26	8.67	−0.3	0.38	−0.21	0.12	E2	
		qOC-12-2	ICIM-3	12	9,150,278–9,461,950	3.18	10.07	0.23	0.13	−0.13	−0.22	E5	
		qOC-13-1	ICIM-3	13	2,267,617–17,851,239	3.98	9.97	−0.14	0.2	−0.25	0.19	E4	Qi et al., 2011a
		qOC-13-3	ICIM-3	13	5,680,211–36,010,071	3.35	7.59	−0.18	−0.07	0.37	−0.12	E4	Qi et al., 2011a; Eskandari et al., 2013; Wang et al., 2014b
		qOC-14-4	ICIM-3	14	8,780,886–16,178,681	3.25	9.2	0.01	0	0.22	−0.23	E5	Tajuddin et al., 2003; Qi et al., 2011a; Rossi et al., 2013
		qOC-15-1	ICIM-3	15	12,793,869–12,867,460	3.07	11.95	−0.16	0.07	−0.19	0.28	E3	Chen et al., 2007
		qOC-18-1	ICIM-3/ICIM-P	18	3,253,447–3,422,683	5.3	8.89/9.16	0.21/0.21	−0.07/−0.07	0.13/0.13	−0.28/−0.28	E1	
						3.36	7.5	0.28	−0.02	0.14	−0.39	E2	
		qOC-19-1	ICIM-3	19	44,754,259–45,487,455	3.27	11.26	0.24	0.02	−0.26	0	E3	Hyten et al., 2004
		qOC-19-2	ICIM-3	19	48,060,796–48,698,594	3.4	7.01	0.25	−0.06	−0.21	0.01	E4	
OC	D2	qOC-1-3	ICIM-3/ICIM-P	1	36,970,568–40,053,928	8.47	15.84/10.94	−0.44/−0.44	0.26/0.26	−0.14/−0.14	0.31/0.31	E4	Hyten et al., 2004; Qi et al., 2011a
		qOC-1-4	ICIM-3	1	51,928,667–52,201,376	3.57	9.73	0.17	0.11	0.11	−0.4	E3	Eskandari et al., 2013
		qOC-5-1	ICIM-3	5	25,952,089–26,680,891	3.06	6.87	−0.06	−0.1	0.25	−0.09	E1	Mansur et al., 1996; Qi et al., 2011a; Rossi et al., 2013; Han et al., 2015
						3.2	9.09	−0.28	−0.1	0.42	−0.04	E2	
		qOC-5-3	ICIM-3	5	32,194,294–34,580,644	4.11	14.74	−0.01	−0.12	0.36	−0.23	E1	Lark et al., 1994; Brummer et al., 1997
		qOC-7-3	ICIM-3	7	39,411,702–40,613,918	3	4.77	0.18	0.03	−0.26	0.05	E4	Tajuddin et al., 2003
		qOC-8-2	ICIM-3	8	3,769,173–4,265,916	3.32	5.24	−0.13	−0.03	−0.14	0.29	E4	
		qOC-9-2	ICIM-3	9	42,151,295–44,909,287	4.27	8.43	0.19	0.2	−0.36	−0.03	E4	
		qOC-9-3	ICIM-3	9	45,813,871–46,489,391	3.43	5.14	0.05	−0.19	0.01	0.13	E5	Brummer et al., 1997; Qi et al., 2011a; Wang et al., 2014b
		qOC-10-2	ICIM-3/ICIM-P	10	25,630,073–46,318,336	5.82	9.14/11.51	0.12/0.12	−0.18/−0.18	−0.13/−0.13	0.19/0.19	E5	Mao et al., 2013
		qOC-11-1	ICIM-3	11	13,537,525–29,892,459	4.64	11.02	0.4	−0.23	−0.26	0.09	E4	Qi et al., 2011a; Han et al., 2015
		qOC-12-1	ICIM-3	12	4,850,009–5,063,075	4.64	13.18	−0.12	−0.3	0.49	−0.06	E2	Brummer et al., 1997; Leite et al., 2016
		qOC-13-2	ICIM-3	13	30,354,037–30,651,747	4.21	6.47	0.11	−0.08	0.14	−0.17	E5	
		qOC-14-1	ICIM-3	14	13,471,993–46,263,682	3.79	5.79	−0.12	0.17	−0.12	0.07	E5	Csanádi et al., 2001; Tajuddin et al., 2003; Chen et al., 2007; Liang et al., 2010; Qi et al., 2011a; Eskandari et al., 2013; Mao et al., 2013; Han et al., 2015
		qOC-14-2	ICIM-3	14	43,482,237–47,439,647	3.43	11.27	0.04	−0.43	0.22	0.18	E3	Chen et al., 2007; Liang et al., 2010; Eskandari et al., 2013; Mao et al., 2013
		qOC-14-3	ICIM-3	14	48,459,883–48,566,172	4.27	13.76	−0.26	0.32	0.22	−0.28	E3	Gai et al., 2007
		qOC-15-1	ICIM-3	15	12,793,869–12,867,460	4.29	16.95	−0.27	0.29	−0.34	0.32	E3	Chen et al., 2007
		qOC-15-2	ICIM-3	15	50,955,346–51,050,706	4.35	6.8	−0.1	−0.17	0.15	0.12	E5	Li et al., 2011
		qOC-17-1	ICIM-3	17	188,378–36,960,374	4.23	7.29	−0.14	−0.15	0.2	0.09	E5	Hyten et al., 2004
		qOC-17-2	ICIM-3	17	37,832,083–38,354,417	3.14	5.61	−0.17	−0.18	0.15	0.2	E4	Mao et al., 2013
		qOC-18-2	ICIM-3/ICIM-P	18	2,689,630–3,041,105	8.87	14.94/18.81	0.32/0.32	0.08/0.08	−0.22/−0.22	−0.18/−0.18	E5	

a *D1: the first (lower) planting density (2.15 × 105 plants ha^−1^), D2: the second (higher) planting density (3 × 105 plants ha^−1^)*.

b *PVE, phenotypic variation explained*.

c *Add1, Add2, Add3, Add4: additive effects from Kenfeng14, Kenfeng15, Heinong48, Kenfeng19, respectively. Additive effect over than 0.05% are positive alleles*.

d *E1: Harbin in 2015; E2: Keshan in 2015; E3: Acheng in 2016; E4: Shuangcheng in 2016; E5: Harbin in 2016*.

**Figure 8:**
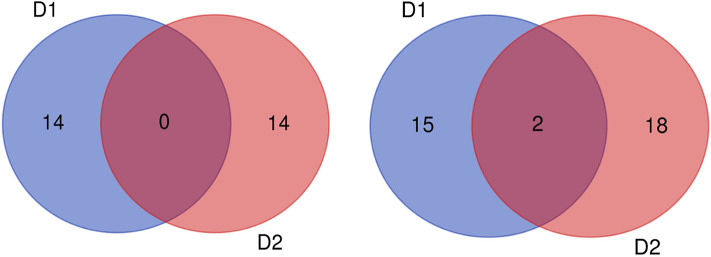
Number of QTLs for protein (left) and oil (right) content detected under two planting densities. D1: the first (lower) planting density (2.15 × 10^5^ plants ha^−1^), D2: the second (higher) planting density (3 × 10^5^ plants ha^−1^).

Of the QTLs detected, one protein-content QTLs (qPC-18-2) and three oil-content QTLs (qOC-18-1, qOC-10-1, qOC-5-1) were detected in more than two environments with PVE of 6.87–9.16%, while the 27 remaining protein-content QTLs and 34 oil-content QTLs were detected in specific environments (D1: five PC QTLs and two OC in E1, three PC QTLs and two OC QTLs in E2, two PC QTLs and two OC QTLs in E3, two PC QTLs and five OC QTLs in E4, one PC QTLs and four OC QTLs in E5; D2: two PC QTLs and one OC QTLs in E1, one OC QTLs in E2, six PC QTLs and four OC QTLs in E3, two PC QTLs and six OC QTLs in E4, four PC QTLs and seven OC QTLs in E5). These results indicate that the genetic basis for protein and oil content differed in various environments (Figure 8).

Of the QTLs detected, there were 19, 9, 14 and 9 positive alleles that enhanced protein content in Kenfeng 14, Kenfeng 15, Heinong 48 and Kenfeng 19, respectively, and 15, 15, 17 and 19 positive alleles that enhanced oil content in Kenfeng14, Kenfeng15, Heinong48 and Kenfeng19, respectively. Therefore, these QTLs increased the protein and oil contents in the soybean plants investigated (Figure S1).

### QTLs for Response to Density

**Figure 9:**
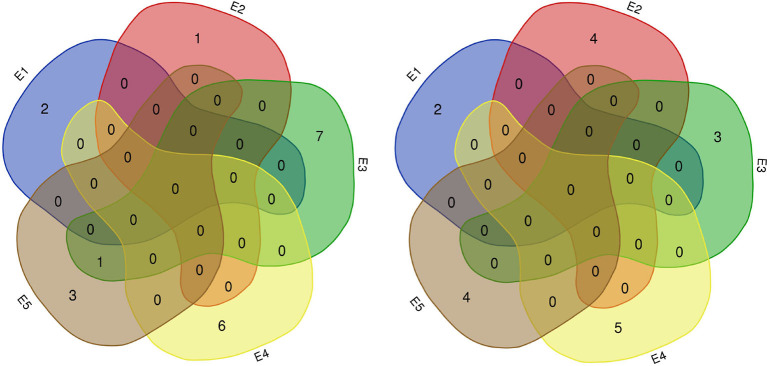
Number of RD QTLs for protein (left) and oil (right) content detected in five environments. E1: Harbin (2015), E2: Keshan (2015), E3: Acheng (2016), E4: Shuangcheng (2016), E5: Harbin (2016).

**Figure 10:**
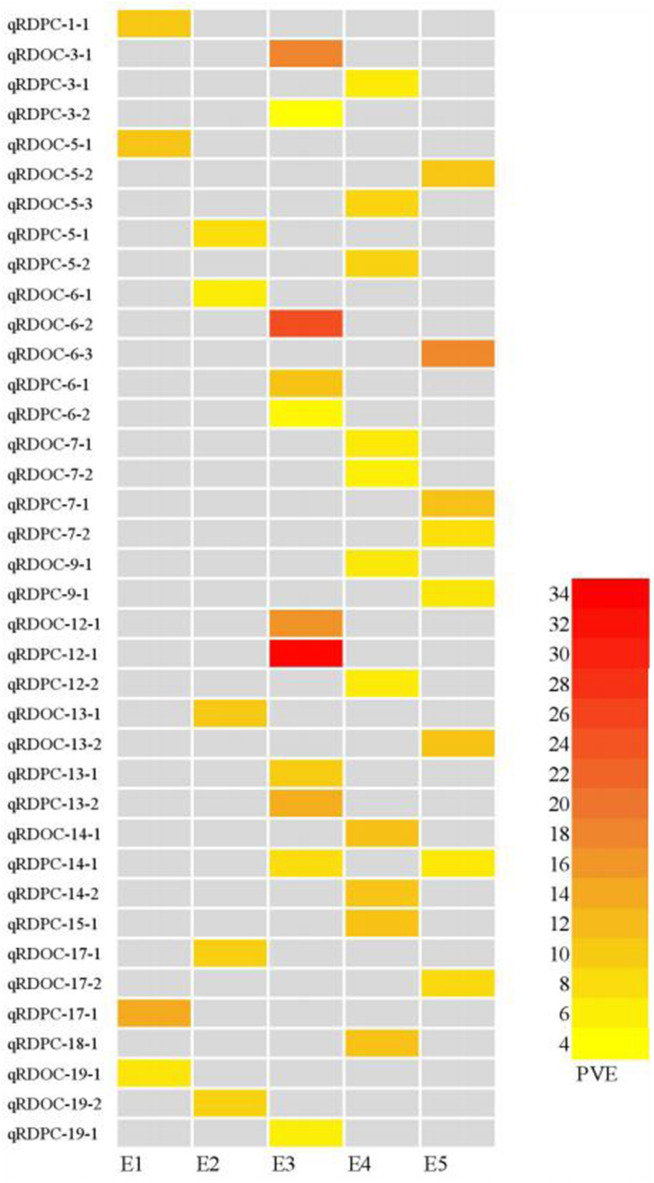
Heatmap of QTLs for the responses to density difference with PVE values higher than 10%. E1: Harbin in 2015; E2: Keshan in 2015; E3: Acheng in 2016; E4: Shuangcheng in 2016; E5: Harbin in 2016.

We identified 38 QTLs for the response of protein and oil content to density (RD) (Figures 3, 4) under LOD value of 3 and under permutation method, which 5 RDPC QTLs were located in two method. Briefly, 20 RD QTLs for protein content were identified on various chromosomes, including Chr01, Chr03, Chr05, Chr06, Chr07, Chr09, Chr12, Chr13, Chr14, Chr15, Chr17, Chr18, and Chr19, with LOD values ranging from 3.05 to 13.68 and accounting for a phenotypic variance of 3.64% (qRDPC-3-2)-38.02% (qRDPC-12-1). Moreover, 18 QTLs for oil content were located on 10 different chromosomes. The PVE values of single QTLs for oil content ranged from 5.88% (qRDOC-7-2) to 24.68% (qRDOC-6-2), with a LOD value ranging from 3.04 to 4.85 (Table 6). There were 19 RD QTLs with a PVE >10%, indicating that the RD trait is controlled by both major-effect and minor-effect QTLs (Figure 9). Among the RD QTLs, one QTL for protein content were detected in two environments, and the 19 remaining RD QTLs for protein content and 18 for oil content were environment specific (Figure 10). The finding that not all RD QTLs were detected in all environments might explain the differences in protein and oil contents in response to planting density.

**Table 6:** RD QTLs for protein and oil content under different planting densities.

**QTL^a^**	**Method**	**Chromosome**	**Physical position (bp)**	**LOD**	**PVE (%)^b^**	**Add1^c^**	**Add2^c^**	**Add3^c^**	**Add4^c^**	**Environment^d^**	**References**
qRDPC-1-1	ICIM-3	1	41,354,793–43,754,056	3.16	10.3	−0.22	−0.42	0.14	0.5	E1	
qRDPC-3-1	ICIM-3	3	1,393,672–1,560,122	3.13	6.44	0.21	0.17	0.17	−0.55	E4	
qRDPC-3-2	ICIM-3	3	3,053,942–3,250,514	3.1	3.64	−0.03	−0.35	0.69	−0.32	E3	
qRDPC-5-1	ICIM-3	5	3,894,485–32,350,213	3.14	7.86	−0.19	−0.68	0.43	0.44	E2	Mansur et al., 1996; Mao et al., 2013
qRDPC-5-2	ICIM-3	5	30,826,149–40,096,192	3.05	9.1	0.42	−0.43	0.44	−0.43	E4	Jun et al., 2008; Pathan et al., 2013
qRDPC-6-1	ICIM-3/ICIM-P	6	47,553,280–47,860,433	6.71	10.87/12.42	0.93/0.93	0.47/0.47	−0.76/−0.76	−0.64/−0.64	E3	Csanádi et al., 2001; Hyten et al., 2004
qRDPC-6-2	ICIM-3	6	49,930,570–50,252,758	3.24	5.06	−1.28	0.31	0.6	0.37	E3	
qRDPC-7-1	ICIM-3/ICIM-P	7	1,974,268–2,303,275	6.28	10.92/13.12	−0.66/−0.66	0.53/0.53	0.68/0.68	−0.55/−0.55	E5	Mao et al., 2013
qRDPC-7-2	ICIM-3	7	451,458–511,687	4.76	7.61	0.27	−0.72	−0.04	0.49	E5	
qRDPC-9-1	ICIM-3	9	4,674,753–14,751,759	3.41	7.04	−0.85	0.22	0.35	0.28	E5	Specht et al., 2001
qRDPC-12-1	ICIM-3/ICIM-P	12	16,850,384–16,953,996	13.68	33.26/38.02	1.15/1.15	−1.44/−1.14	−0.79/−0.79	1.08/1.08	E3	
qRDPC-12-2	ICIM-3	12	3,175,045–4,701,685	3.14	6.37	−0.25	−0.15	0.64	−0.23	E4	Liang et al., 2010
qRDPC-13-1	ICIM-3/ICIM-P	13	15,750,139–16,469,296	5.94	9.98/11.41	0.42/0.42	−0.78/−0.78	−0.34/0.34	0.71/0.71	E3	Mao et al., 2013
qRDPC-13-2	ICIM-3/ICIM-P	13	444,838–43,052,819	7.73	13.36/15.27	−1.03/−1.03	0.74/0.74	−0.41/−0.41	0.7/0.7	E3	Mao et al., 2013
qRDPC-14-1	ICIM-3	14	2,256,954–4,780,532	3.55	8.06	0.52	−1.09	0.21	0.37	E3	Akond et al., 2014
				3.76	6.62	0.68	0.16	−0.24	−0.6	E5	
qRDPC-14-2	ICIM-3	14	4,450,691–4,558,092	4.67	10.74	0.62	−0.36	0.32	−0.58	E4	Akond et al., 2014
qRDPC-15-1	ICIM-3	15	51,351,343–51,546,757	4.96	10.87	0.4	0.43	−0.02	−0.81	E4	
qRDPC-17-1	ICIM-3	17	39,258,351–39,557,300	4.28	13.73	0	−0.41	−0.23	0.64	E1	Tajuddin et al., 2003; Warrington et al., 2015
qRDPC-18-1	ICIM-3	18	12,546,166–55,369,435	3.67	10.93	0.33	−0.34	0.58	−0.58	E4	Diers et al., 1992; Brummer et al., 1997; Jun et al., 2008; Liang et al., 2010; Lu et al., 2013; Mao et al., 2013
qRDPC-19-1	ICIM-3	19	47,453,417–47,560,466	3.8	6.04	0.5	0.4	−0.26	−0.64	E3	
qRDOC-3-1	ICIM-3	3	31,574,922–31,782,255	3.81	18.13	−0.27	0.82	−0.21	−0.34	E3	Mao et al., 2013
qRDOC-5-1	ICIM-3	5	32,194,294–34,580,644	3.24	10.79	−0.02	−0.09	0.32	−0.21	E1	Lark et al., 1994; Brummer et al., 1997
qRDOC-5-2	ICIM-3	5	35,250,550–35,462,154	3.36	10.53	−0.12	0.22	−0.18	0.07	E5	
qRDOC-5-3	ICIM-3	5	37,674,734–38,453,062	3.38	8.89	0.12	0.21	−0.76	0.43	E4	
qRDOC-6-1	ICIM-3	6	49,304,240–49,371,230	3.07	6.25	−0.04	0.05	−0.22	0.21	E2	
qRDOC-6-2	ICIM-3	6	50,554,375–50,757,507	4.2	24.68	−0.03	0.26	0.17	−0.4	E3	
qRDOC-6-3	ICIM-3	6	7,359,493–7,951,010	4.85	17.67	−0.32	0.12	0.25	−0.05	E5	Pathan et al., 2013
qRDOC-7-1	ICIM-3	7	39,411,702–40,613,918	3.23	6.55	0.08	0.2	−0.38	0.09	E4	Tajuddin et al., 2003
qRDOC-7-2	ICIM-3	7	40,281,053–41,513,649	3.36	5.88	−0.32	0.12	−0.11	0.32	E4	Tajuddin et al., 2003
qRDOC-9-1	ICIM-3	9	42,151,295–44,909,287	3.1	6.75	0.16	0.25	−0.4	−0.01	E4	Mao et al., 2013
qRDOC-12-1	ICIM-3	12	1,650,694–1,753,755	3.34	16.42	−0.13	0.34	−0.24	0.03	E3	Leite et al., 2016
qRDOC-13-1	ICIM-3	13	21,697,193–24,456,483	3.27	10.3	0.19	0.02	−0.37	0.16	E2	
qRDOC-13-2	ICIM-3	13	29,667,064–30,760,581	3.95	10.89	0.2	0.01	0.04	−0.24	E5	
qRDOC-14-1	ICIM-3	14	42,848,816–45,178,466	3.44	11.16	−0.54	0.21	0.06	0.27	E4	Tajuddin et al., 2003; Chen et al., 2007; Liang et al., 2010; Qi et al., 2011b; Eskandari et al., 2013; Mao et al., 2013
qRDOC-17-1	ICIM-3	17	11,051,716–12,950,239	3.38	9.46	0.28	−0.17	0.11	−0.23	E2	Lee et al., 1996; Qi et al., 2011b
qRDOC-17-2	ICIM-3	17	13,950,712–14,050,896	3.04	8.39	0.23	−0.2	0.03	−0.07	E5	Lee et al., 1996
qRDOC-19-1	ICIM-3	19	20,050,036–33,323,501	3.12	6.94	−0.11	0.2	0.09	−0.18	E1	
qRDOC-19-2	ICIM-3	19	44,675,241–45,395,438	3.68	9.09	0.26	0.11	−0.22	−0.15	E2	Hyten et al., 2004

a *RD. Density effect*.

b *PVE, phenotypic variation explanation ratio*.

c *Add1, Add2, Add3, Add4: additive effects from Kenfeng14, Kenfeng15, Heinong48, Kenfeng19. Additive effect over than 0.05% are positive alleles*.

d *E1: Harbin in 2015; E2: Keshan in 2015; E3: Acheng in2016; E4: Shuangcheng in 2016; E5: Harbin in 2016*.

Among the RD QTLs, 11, 8, 12 and 9 positive alleles from Kenfeng14, Kenfeng15, Heinong48 and Kenfeng19, respectively increased the protein content at higher planting density. Similarly, 8, 13, 6 and 7 positive alleles from Kenfeng14, Kenfeng15, Heinong48 and Kenfeng19, respectively increased the oil content when the density increased from D1 to D2 (Figure S1).

### Analysis of Potential Candidate Genes

**Figure 11:**
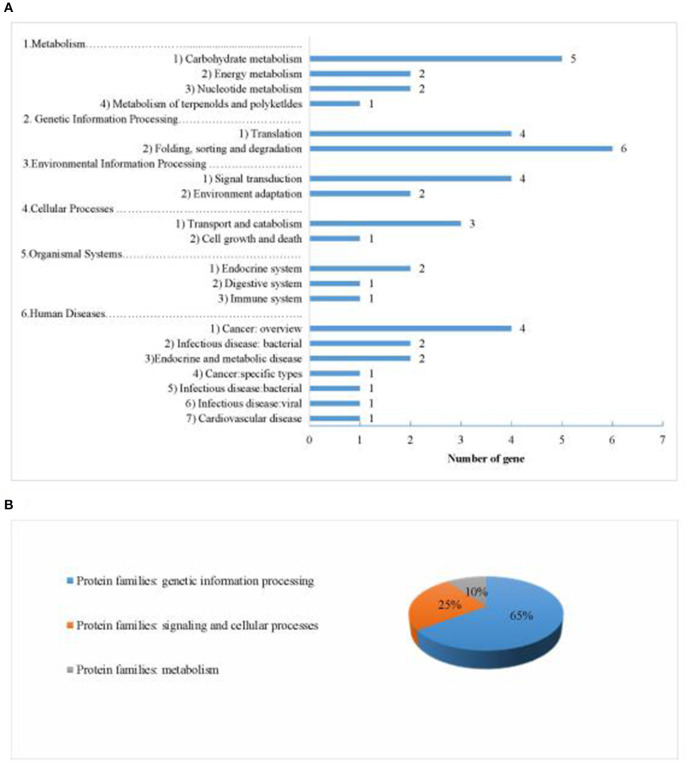
Information about the pathways and orthologous protein families of the 26 annotated candidate genes. **(A)** shows information about the pathways. **(B)** shows information about the orthologous protein families.

We identified 484 genes based on 23 QTLs under different planting densities and with the responses to density difference. Seventy-six genes were highly expressed within these regions in seeds, with 26 annotated genes in 20 pathways and 3 protein families identified in the KEGG database (Figure 11). Among these, four genes were selected as potential candidate genes affecting protein and oil content due to their annotations and functions in various metabolic pathways (Table 7; see bold text).

**Table 7:** Details about the 26 genes annotated for protein and oil content in the KEGG database.

**QTL name**	**Chromosome**	**Gene name**	**Position**	**KO number**	**Annotation**
**qPC-3-1^a^**	**3**	**Glyma.03G014600.1**	**1,465,953–1,473,744**	**K00028**	**Malate dehydrogenase (decarboxylating) [EC:1.1.1.39]**
qPC-3-1	3	Glyma.03G014300.1	1,427,595–1,433,362	K15095	(+)-neomenthol dehydrogenase [EC:1.1.1.208]
qRDOC-5-2	5	Glyma.05G161700.1	35,312,249–35,316,256	K10949	ER lumen protein retaining receptor
qRDPC-6-1	6	Glyma.06G289400.1	47,813,934–47,821,512	K03028	26S proteasome regulatory subunit N1
qPC-10-3	10	Glyma.10G217600.1	44,929,764–44,934,766	K09580	Protein disulfide-isomerase A1 [EC:5.3.4.1]
qPC-10-3	10	Glyma.10G218700.1	45,056,903–45,059,806	K19476	Vacuolar protein sorting-associated protein IST1
**qPC-10-3^a^**	**10**	**Glyma.10G215400.1**	**44,744,151–44,748,536**	**K00627**	**Pyruvate dehydrogenase E2 component (dihydrolipoamide acetyltransferase) [EC:2.3.1.12]**
qPC-10-4	10	Glyma.10G223400.1	45,440,350–45,443,915	K02925	Large subunit ribosomal protein L3e
qPC-10-4	10	Glyma.10G221600.1	45,320,219–45,322,143	K02964	Small subunit ribosomal protein S18e
		Glyma.10G224200.1	45,498,206–45,500,186		
qPC-10-4	10	Glyma.10G222900.1	45,393,914–45,399,095	K16675	Palmitoyltransferase ZDHHC9/14/18 [EC:2.3.1.225]
qPC-10-4	10	Glyma.10G221500.1	45,294,737–45,316,113	K12124	GIGANTEA
qPC-10-4	10	Glyma.10G220800.1	45,207,565–45,210,349	K15306	Ran-binding protein 1
qOC-12-1	12	Glyma.12G066700.1	9,170,707–9,174,178	K02893	Large subunit ribosomal protein L23Ae
qOC-12-1	12	Glyma.12G067700.1	4,960,025–4,964,029	K09580	Protein disulfide-isomerase A1 [EC:5.3.4.1]
qOC-12-2	12	Glyma.12G102900.1	9,170,707–9,174,178	K01177	Beta-amylase [EC:3.2.1.2]
qPC-12-1	12	Glyma.12G139000.1	16,937,219–16,942,610	K08202	MFS transporter, OCT family, solute carrier family 22 (organic cation transporter), member 4/5
qOC-14-3	14	Glyma.14G219700.1	48,476,500–48,481,440	K09487	Heat shock protein 90kDa beta
qOC-14-3	14	Glyma.14G220300.1	48,529,968–48,538,718	K18468	Vacuolar protein sorting-associated protein 35
qRDPC-14-2	14	Glyma.14G056900.1	4,549,808–4,552,551	K02987	Small subunit ribosomal protein S4e
qRDPC-14-2	14	Glyma.14G056800.1	4,541,231–4,543,915	K00522	Ferritin heavy chain [EC:1.16.3.2]
qOC-18-1	18	Glyma.18G036300.1	2,840,498–2,844,892	K01952	Phosphoribosylformylglycinamidine synthase [EC:6.3.5.3]
qOC-18-1	18	Glyma.18G035000.1	2,727,985–2,730,047	K14497	Protein phosphatase 2C [EC:3.1.3.16]
qOC-18-1	18	Glyma.18G035700.1	2,772,168–2,779,256	K18443	Golgi-specific brefeldin A-resistance guanine nucleotide exchange factor 1
		Glyma.18G035800.1	2,781,077–2,787,829		
**qOC-19-1^a^**	**19**	**Glyma.19G190100.1**	**44,772,176–44,779,340**	**K00873**	**Pyruvate kinase [EC:2.7.1.40]**
**qOC-19-1^a^**	**19**	**Glyma.19G190900.1**	**44,841,863–44,846,599**	**K01689**	**Enolase [EC:4.2.1.11]**
qOC-19-1	19	Glyma.19G191100.1	44,848,456–44,851,043	K12836	Splicing factor U2AF 35 kDa subunit

a *Bold font indicates the genes relating with the protein and oil anabolism in soybean according to our deduction*.

## Discussion

### QTLs With Effects on Protein and Oil Content in Soybean at Different Planting Densities

Protein and oil contents are quantitative traits that are affected by environmental conditions. Competition for nutritional resources occurs when the planting density changes, and different plant varieties can respond differently. In the current study, the protein content of four soybean varieties and the oil content of five soybean varieties increased when the planting density increased from D1 to D2 in five environments (Figure 2). However, the responses of the remaining varieties to density were inconsistent, indicating that the environment affects the expression of genes that control protein and oil content. Bellaloui et al. (2014) used four soybean varieties to study the effects of planting density on protein and oil content. The protein contents of soybean varieties P93M90, AG 3906, P94B73, and V52N3 reached their maximum levels at a planting density of 518,700, 518,700, 180,000, and 150,000 plants ha^−1^, respectively. Similarly, the oil contents of the four varieties were highest at a planting density of 247,000, 444,600, 150,000, and 60,000 plants ha^−1^, respectively. These results indicate that the responses of individual protein and oil contents vary depending on the planting density. Therefore, the results of the present study are in agreement with those reported by Bellaloui et al. (2014).

Similar to the physiological responses to abiotic stresses, such as water deficiency, waterlogging, low phosphorus levels, cold temperatures, and light and nitrogen deficiency, a specific molecular mechanism also controls the responses of protein and oil content to increasing planting density (Osman et al., 2013). There are two aspects of the genetic basis of the effects of QTLs on the variation of protein and oil content at increasing planting density. First, the cumulative effects of QTLs could be detected directly based on protein and oil contents at a specific density in a particular environment. Here, these phenotypic values reflect the cumulative effects of genotype, macro-environment (location and years), interactions between genotype and environment, and planting density. The results of QTL mapping of the same trait can vary in different environments. Ku et al. (2015) evaluated the effects of two different planting densities (60,000 and 120,000 plant/hm^2^) on three plant height-related traits (plant height, ear height, and ear height-to-plant height ratio) in maize. Nine QTLs were detected at the low planting density, and seven QTLs were detected at the high planting density. Akond et al. (2012) used a RIL population derived from a cross between soybean lines PI 438489B and Hamilton to detect QTLs for PC and OC under two planting densities. Three QTLs for protein content were detected at a low planting density (50 cm row space), while 2 QTLs for protein content and 6 QTLs for oil content were detected at a high planting density (25 cm row space). In the current study, we used an FW-RIL population to detect density-specific QTLs for protein and oil content. We identified 28 protein-content QTLs and 35 oil-content QTLs at planting densities D1 and D2, respectively. Among these QTLs, two QTLs was identified at both planting densities, whereas the remaining QTLs were detected at different densities. These results indicate that the genetic basis for the cumulative effects of QTLs on protein and oil content under two densities strongly differed, as indicated by significant genotype by density interaction effects (*P* ≤ 0.01; Table 2).

Second, the net effects of QTLs on planting density could be identified based on increases in protein and oil content in response to density. To analyze the net effect of changes in planting density, the effects of all factors except planting density must be removed. Zhu (1995) proposed a conditional variable method to exclude the background from the covariance of related traits, allowing the net effects of some factors to be identified. This method has been used to estimate the net effects of various developmental stages (Xue et al., 2019) and correlated traits (Li et al., 2020). Here, we estimated the responses of protein and oil content to planting density using this method. We performed QTL mapping for the effect of increased planting density from 2.15 × 10^5^ plants/ha (D1) to 3 × 10^5^ plants/ha (D2) on protein and oil content. Using linkage analysis, we identified 20 and 18 RD QTLs controlling the responses of soybean protein and oil content to density, respectively, indicating that a specific molecular mechanism regulates the response to increasing planting density from D1 to D2. Therefore, when high-quality soybean varieties suitable for high-density planting were bred, alleles with positive effects should be pyramided. Conversely, when high-quality soybean varieties suitable for low-density planting was selected, alleles with negative effects should be combined.

### Potential Candidate Genes Associated With Protein and Oil Contents

Based on the QTLs detected under different planting densities and the responses to density difference, as well as their pathway annotations, we identified four genes that might be related to differences in protein and oil contents under different plant densities (Table 6; see bold text).

Energy produced in plants via photosynthesis is stored in the form of proteins, lipids, and other organic compounds. *Glyma.10G215400.1* encodes pyruvate dehydrogenase E2 component, is mainly involved in carbon metabolism, citrate cycle (TCA cycle), and glycolysis/gluconeogenesis. This enzyme catalyzes the formation of pyruvate, which is the main substrate for the Calvin cycle, so we believe that the gene is affected by planting density, and closely related to protein and oil content. *Glyma.19G190100.1* encodes an enzyme that regulates the formation of pyruvate kinase (PK) and plays an important role in carbon dioxide fixation and glycolysis in chloroplasts under light stimulation (Grodzinski et al., 1999). The enzyme participates in the glycolysis pathway, and metabolites can provide a premise for acetyl-CoA to form fatty acids and provide a carbon skeleton for fatty acid synthesis (Ambasht and Kayastha, 2002; Sébastien et al., 2007). Sucrose and starch produced by the glycolysis pathway can promote mitochondrial respiration and the TCA cycle, The TCA cycle is a key metabolic pathway for the oxidation of sugars, fats, and proteins (Robinson et al., 1991; Horchani et al., 2010). Therefore, we believe that this enzyme affects the protein and oil contents of soybean. *Glyma.03G014600.1* encodes malate dehydrogenase (MDH), an important enzyme that catalyzes malic acid formation and is significantly affected by light stimulation, it is photo regulatory enzyme (Li et al., 1999). Malate is an important intermediate metabolite in plant cells, which are many biological functions in metabolic pathways (glyoxylate cycle, tricarboxylic acid cycle, glucose synthesis, amino acid synthesis, and redox stability), we know that these metabolic pathways are related to protein and oil synthesis (Minarik et al., 2002; Matsuda et al., 2010). Moreover, this enzyme is a key enzyme in the C4 pathway in Wheat (Hata and Matsuoka, 1987) and Soybean (Hao et al., 1991), which maintain a high photosynthetic carbon assimilation capacity when the light capacity and carbon dioxide content decrease. The activity of this enzyme is affected by oxygen concentration. Changes in planting density affect the oxygen concentration around plants, and changes in the expression of *Glyma.03G014600.1* affect photosynthesis and the synthesis of proteins and oils. Therefore, changes in planting density cause competition between plants, which affect the ability of plants to absorb light, as well as oxygen. Therefore, we conclude that the gene responsible for this enzyme plays a role in regulating protein and oil contents in soybean. *Glyma.19G190900.1* encodes an enzyme that catalyzes the enolase, which is involved in the changes in protein and oil content resulting from change of temperature. The activity of enolase continues to increase after temperature change, which strengthen the glycolysis process, thus promoting plant growth (Thomashow, 1999). Changes in planting density result in temperature in different plant varieties. Increasing the activity of enzymes that promote the glycolysis pathway process will enhance the protein and oil contents of plants. These findings could be used to enhance the protein and oil contents of soybean in the future.

### Comparison of QTL Mapping Using LOD Value Is 3 and Permutation Method

LOD (log of odd) threshold is usually used to assure the existence of QTL. There are two methods to choose LOD threshold according to research goal for detect QTL. The first method is by permutation tests (Doerge and Churchill, 1996) which will generate a higher LOD threshold to decrease false positive QTL in each detection procedure. By this method a small amount of major effect QTL could be detected (Panthee et al., 2005; Bachlava et al., 2009; Tucker et al., 2010). The shortage of this method is consumption of time in the larger amount of calculation, and even failure in a large genome data. Sun et al. (2013) propose a method to certain the LOD threshold in QTL mapping, based on the genome-wide significance level, the population type, marker density and genome size, and Zhang P. et al. (2017) specified the threshold LOD value 3.766 for ICIM and IM of four-way cross recombinant inbred lines population to control the genome-wide typed one error at an equal level of 0.05. The second way is to set a lower LOD threshold (LOD = 2.0, equivalently *P* = 0.002 and significant at *P* < 0.01) with objective to avoid missing of QTLs due to slightly lower significance and the putative QTL were assured by repeated detected in multiple environments or multiple genetic background (Cornelious et al., 2005; Yesudas et al., 2013; Wang et al., 2014a,b). In this research, we firstly detected QTL depending on LOD threshold generated by permutation (1,000 repeats and 0.05 type one error probability), and 5, 6, 5 QTLs for PC, OC, RDPC with higher PVE (7.19%-38.02%) were detected. Among the 16 QTLs, only qOC-1-1 was repeatedly identified in E1 and E2.The major QTL could be detected directly by permutation which will miss minor QTL. The detection of minor QTL is necessary the inheritance and breeding of quality traits under multiple environments. The comparison on minor QTL detected under multiple environments and plant densities could explain the difference of genetic basis (molecular ecotypes). Furthermore, the accumulation of minor QTL could increase prediction of protein and oil content under some single planting density. Aimed to screen common and specific QTL under various densities, considering our linkage map characters (there are 1,031 marker intervals in 20 chromosome of 3539.66 cM length) (Table S3), we choose a lower LOD threshold 3 (equivalently genome-wise *P* = 0.005) to screen QTL in the whole genome by ICIM. A total of 23, 31, 15 and 18 QTLs controlling PC, OC, RDPC, and RDOC with lower PVE (3.64–24.68%) were added. Then, five (qOC-1-1, qOC-5-1, qOC-10-1, qPC-18-2, and qRDPC-14-1) and two (qOC-15-1 and qOC-7-3) of all 103 QTLs were detected repeatedly in in two environment and two planting densities, respectively. Comparing two kinds of LOD threshold, five QTLs (qOC-10-1, qOC-15-1, qOC-5-1, qOC-7-3, qRDPC-14-1) could be verified the facticity by repeated identification under lower LOD threshold. In summarization, the permutation is suitable to detect the major QTL for higher PVE, and the repeated identification in multiple environments under lower threshold is feasible to discover QTL with low PVE.

### Comparison of the Present Study With Previous Research

In present study, we identified 65 QTLs for protein and oil content under different planting densities and 38 QTLs with the responses to density difference. Using the SoyBase database (http://www.soybase.org/), we concluded that 70 QTLs were in agreement with previous reports, including 29 QTLs that overlapped with marker intervals identified in previous studies (Lark et al., 1994; Mansur et al., 1996; Brummer et al., 1997; Chapman et al., 2003; Tajuddin et al., 2003; Hyten et al., 2004; Stombaugh et al., 2004; Reinprecht et al., 2006; Chen et al., 2007; Gai et al., 2007; Liang et al., 2010; Qi et al., 2011b; Lu et al., 2013; Mao et al., 2013; Pathan et al., 2013; Rossi et al., 2013; Akond et al., 2014; Wang et al., 2014b; Han et al., 2015; Warrington et al., 2015). More importantly, the PVE for 29 QTLs was >10%. The significance of these regions was used to identify genes responsible for the increased protein and oil contents of soybean under increased planting density.

The remaining QTLs that are consistent with previously reported QTLs for protein and oil content are listed in Tables 3–5. Among the QTLs detected, 33 novel QTLs (qPC-1-1, qPC-5-2, qPC-10-4, qPC-3-1, qPC-5-1, qPC-8-2, qPC-12-1, qPC-12-2, qPC-14-1, qOC-7-1, qOC-10-1, qOC-12-2, qOC-18-2, qOC-19-2, qOC-8-2, qOC-9-2, qOC-13-2, qOC-18-1, qRDPC-1-1, qRDPC-3-1, qRDPC-3-2, qRDPC-6-2, qRDPC-7-2, qRDPC-12-1, qRDPC-15-1, qRDPC-19-1, qRDOC-5-2, qRDOC-5-3, qRDOC-6-1, qRDOC-6-2, qRDOC-13-1, qRDOC-13-2, qRDOC-19-1) were identified. These QTLs require further verification before they can be used in breeding programs.

## Summary

In this study, we identified 65 QTLs for protein and oil content under different planting densities and 38 QTLs with density responses based on SNP mapping. Based on these QTLs, four candidate genes were identified: these genes are affected by planting density and control protein and oil content. The molecular mechanism for the formation of protein and oil content under multiple planting densities involves the cumulative effects of QTLs and the response to increases in planting density. These findings lay the foundation for enhancing protein and oil contents and increasing yields in soybean at specific planting densities.

## Data Availability Statement

The datasets GENERATED for this study can be found in Figshare: https://figshare.com/s/4a7b8caea2c29f891bc3.

## Author Contributions

W-XL and HN conceived and designed the experiments. XS, XL, JS, ZQ, YW, YF, JW, SJ, and CY performed the field experiments and quality analysis. SL and ZT performed the genotyping. KZ, XT, and HN analyzed data, drafted and revised the manuscript.

## Conflict of Interest

The authors declare that the research was conducted in the absence of any commercial or financial relationships that could be construed as a potential conflict of interest.

## Supplementary Material

Table S1 Comparison of parental source and quality traits.

Table S2 Planting conditions of the parents and FW-RIL population used for field experiments in five environments.

Table S3 Description of characteristics of 20 chromosomes in the high-density genetic map.

Figure S1 Frequency of positive alleles of QTLs related to protein (left) and oil (right) contents based on their distribution in each parent under different planting densities and in response to density.
