# Supplementary material for: Quantitative Trait Locus Analysis of Protein and Oil Content in Response to Planting Density in Soybean (Glycine max [L.] Merri.) Seeds Based on SNP Linkage Mapping
Source: Front Genet. 2020 Jun 25;11:563. doi: 10.3389/fgene.2020.00563 (PMC7330087; doi:10.3389/fgene.2020.00563)
Supplement: Table S1 — Comparison of parental source and quality traits. [file Table_1.docx]

**Supplementary Table 1.** Comparison of parental source and quality traits

| Variety | Source | Protein content（%） | Oil content  （%） |
| --- | --- | --- | --- |
| Kenfen14 | Suinong 10 × Changnong 5 | 39.69 | 20.34 |
| Kenfen15 | Suinong 14 × Kenjiao 9307 | 38.68 | 22.76 |
| Heinong 48 | Ha 90-6719 × Sui 90-5888 | 44.71 | 19.05 |
| Kenfen19 | Hefeng 25 × (Kefeng 4 ×Gong8861-0) | 42.52 | 19.26 |
