# Supplementary material for: Quantitative Trait Locus Analysis of Protein and Oil Content in Response to Planting Density in Soybean (Glycine max [L.] Merri.) Seeds Based on SNP Linkage Mapping
Source: Front Genet. 2020 Jun 25;11:563. doi: 10.3389/fgene.2020.00563 (PMC7330087; doi:10.3389/fgene.2020.00563)
Supplement: Table S2 — Planting conditions of the parents and FW-RIL population used for field experiments in five environments. [file Table_2.docx]

**Supplementary Table 2.** Planting conditions of the parents and FW-RILs population for field experiments in five environments.

| Environment^a^ | Location | Planting year | Preceding crop | Planting date | Soil type | Available potassium | Available phosphorus | Alkali-hydrolyzable nitrogen | pH | Annual accumulated temperature over 10℃ | Annual rainfall |
| --- | --- | --- | --- | --- | --- | --- | --- | --- | --- | --- | --- |
|  |  |  |  |  |  | (mg·kg^-1^) | (mg·kg^-1^) | (mg·kg^-1^) |  | (℃) | (mm) |
| E1 | Harbin(45°43′N, 126°45′E) | 2015 | Corn | May 7th | Chernozem | 208 ± 24.0 | 51 ± 2.1 | 118 ± 7.2 | 6.5 | >2700 | 423 |
| E2 | Keshan(48°18′N, 126°15′E) | 2015 | Corn | May 10th | Chernozem | 219 ± 32.6 | 55 ± 6.4 | 127.9 ± 7.2 | 6.7 | 2300~2500 | 502.5 |
| E3 | Acheng(45°32′N, 126°58′E) | 2016 | Corn | May 5th | Meadow chernozemic soil | 223 ± 19.7 | 41 ± 5.3 | 102 ± 8.2 | 7.2 | >2700 | 600 |
| E4 | Shuangcheng(45°22′ N, 126°18′E) | 2016 | Corn | May 7th | Meadow chernozemic soil | 243 ± 19.6 | 38 ± 4.4 | 107 ± 9.2 | 6.8 | >2700 | 481 |
| E5 | Northeast Agricultural University, Harbin(45°43′N, 126°45′E) | 2016 | Corn | May 10th | Chernozem | 207 ± 22.2 | 54 ± 3.9 | 123 ± 6.7 | 6.5 | >2700 | 569.1 |

^a^ E1: Haebin in 2015; E2: Keshan in 2015; E3: Acheng in2016; E4: Shuangcheng in 2016; E5: Harbin in 2016.
