# Supplementary material for: Quantitative Trait Locus Analysis of Protein and Oil Content in Response to Planting Density in Soybean (Glycine max [L.] Merri.) Seeds Based on SNP Linkage Mapping
Source: Front Genet. 2020 Jun 25;11:563. doi: 10.3389/fgene.2020.00563 (PMC7330087; doi:10.3389/fgene.2020.00563)
Supplement: Table S3 — Description of characteristics of 20 chromosomes in the high-density genetic map. [file Table_3.docx]

Table S3 Description of characteristics of 20 chromosomes in the high-density genetic map

| Chromosome | Length (cM) | Number of markers | Number of intervals | Number of intervals<5cM | Average of the length of interval | Physical length (pb) | Covering proportion of the physical distance (%) |
| --- | --- | --- | --- | --- | --- | --- | --- |
|  |  |  |  |  |  |  |  |
| 1 | 167.25 | 167 | 59 | 54 | 2.83 | 51021618 | 89.78 |
| 2 | 184.85 | 56 | 40 | 35 | 4.62 | 44702586 | 92.02 |
| 3 | 136.18 | 316 | 71 | 69 | 1.92 | 44160171 | 96.48 |
| 4 | 203.44 | 57 | 33 | 25 | 6.16 | 50442591 | 96.29 |
| 5 | 329.66 | 151 | 73 | 62 | 4.52 | 41744591 | 98.84 |
| 6 | 216.43 | 98 | 58 | 55 | 3.73 | 50682879 | 98.59 |
| 7 | 150.75 | 104 | 53 | 50 | 2.84 | 44546313 | 99.81 |
| 8 | 236.47 | 121 | 71 | 67 | 3.33 | 47520713 | 99.34 |
| 9 | 216.33 | 210 | 78 | 73 | 2.77 | 48982456 | 97.6 |
| 10 | 121.45 | 20 | 13 | 7 | 9.34 | 20688264 | 40.12 |
| 11 | 142.06 | 16 | 13 | 8 | 10.93 | 29938124 | 86.12 |
| 12 | 164.6 | 190 | 65 | 62 | 2.53 | 38927313 | 97.1 |
| 13 | 202.62 | 147 | 79 | 77 | 2.56 | 42607982 | 92.88 |
| 14 | 223.42 | 85 | 49 | 43 | 4.56 | 48941863 | 99.8 |
| 15 | 141.5 | 53 | 36 | 33 | 3.93 | 51440125 | 99.39 |
| 16 | 89.03 | 111 | 32 | 31 | 2.78 | 36250163 | 95.68 |
| 17 | 170.5 | 233 | 74 | 71 | 2.3 | 41363095 | 99.33 |
| 18 | 173.74 | 72 | 47 | 40 | 3.7 | 57096869 | 98.41 |
| 19 | 193.01 | 92 | 65 | 61 | 2.97 | 50126127 | 98.78 |
| 20 | 76.37 | 33 | 22 | 21 | 3.47 | 30127630 | 62.89 |
|  | 3539.66 | 2332 | 1031 |  | 835 |  |  |
