# Supplementary material for: Quantitative Trait Locus Analysis of Protein and Oil Content in Response to Planting Density in Soybean (Glycine max [L.] Merri.) Seeds Based on SNP Linkage Mapping
Source: Front Genet. 2020 Jun 25;11:563. doi: 10.3389/fgene.2020.00563 (PMC7330087; doi:10.3389/fgene.2020.00563)
Supplement: Figure S1 — Frequency of positive alleles of QTLs related to protein (left) and oil (right) contents based on their distribution in each parent under different planting densities and in response to density. [file Presentation_1.zip › Figure S1.DOCX]

**FIGURE S1** | Family tree of soybean four-way recombinant inbred lines population
